# Aberrant expression of cytokeratin in plasma cell neoplasms: A pitfall for diagnostic errors

**DOI:** 10.1002/jha2.574

**Published:** 2022-09-26

**Authors:** Michelle Huang, Ali Sakhdari

**Affiliations:** ^1^ Department of Laboratory Hematology, Toronto General Hospital University Health Network, Toronto, Canada

**Keywords:** Haematological malignancies, immunophenotype, myeloma

1

A 70‐year‐old woman with a previous history of Ig‐Kappa multiple myeloma status post chemotherapy, radiation, and autologous stem cell transplant presented to the hospital with intermittent discomfort and spasmodic pain in bilateral breasts. Breast core biopsies show a diffuse sheet of neoplastic cells with round nuclear contour, open chromatin, eccentric nuclei with distinct nucleolus and relatively abundant eosinophilic cytoplasm (Figure 1A, hematoxylin and eosin). Neoplastic cells show diffuse membranous CD138 (Figure 1B), immunoglobulin (Ig)‐G (Figure 1C) and Kappa light chain (Figure 1D) staining, but not Lambda light chain (Figure 1D, inset) indicative of a monotypic plasma cell population. Interestingly, the neoplastic cells also show diffuse aberrant cytoplasmic staining of AE1/AE3 (Figure 1E), usually a membranous pan‐cytokeratin marker. Upon further investigation, it appears that the neoplastic cells stain positive exclusively with low molecular weight keratins Cam 5.2 (Figure 1F), while negative for high molecular weight keratins CK5/6 (Figure 1F, inset). Here, the plasma cells display an unusual diffuse cytoplasmic cytokeratin staining pattern, which might mislead toward a carcinoma diagnosis. In a few plasma cell myeloma studies, it has been shown that some low molecular weight keratins can join as heteropolymeric filaments to form intracellular scaffolds, suggesting that cytokeratins may play a role in coordinating oncogenic transformation and cellular adhesion [1]. While these results suggest possible functions of cytokeratins in plasma cell neoplasms, one must also be careful in interpreting these staining patterns as it can lead to a potential misdiagnosis.

**FIGURE 1:**
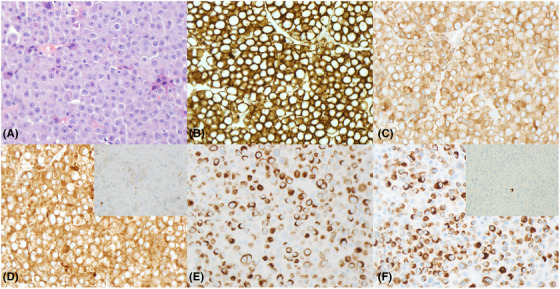
Diffuse sheets of neoplastic plasma cells in multiple myeloma (H&E, A) demonstrating membranous CD138 (B), immunoglobulin (Ig)‐G (C) and Ig‐Kappa (D) positive staining and no Ig‐Lambda (D, inset) staining but with aberrant cytoplasmic AE1/AE3 (E) and Cam5.2 (F) but precluding CK5/6 (F, inset) staining. All magnifications: 400x

## CONFLICT OF INTEREST

The authors declare that they have no conflict of interest.

## FUNDING STATEMENT

The authors received no specific funding for this work.

## ETHICS STATEMENT

This case followed the University Health Nerwork and patients' ethics.
